# *In Vitro* Fermentation of Hyaluronan with Different Molecular Weights by Human Gut Microbiota: Differential Effects on Gut Microbiota Structure and Metabolic Function

**DOI:** 10.3390/polym15092103

**Published:** 2023-04-28

**Authors:** Ruohan Zhao, Chuan Zhang, Leilei Yu, Chengcheng Zhang, Jianxin Zhao, Arjan Narbad, Qixiao Zhai, Fengwei Tian

**Affiliations:** 1 State Key Laboratory of Food Science and Technology, Jiangnan University, Wuxi 214122, China; 2 School of Food Science and Technology, Jiangnan University, Wuxi 214122, China; 3 International Joint Research Laboratory for Probiotics, Jiangnan University, Wuxi 214122, China; 4 Gut Health and Microbiome Institute Strategic Programme, Quadram Institute Bioscience, Norwich NR4 7UA, UK

**Keywords:** hyaluronan, molecular weight, *in vitro* fermentation, gut microbiota, metabolomics

## Abstract

Hyaluronan (HA) has various biological functions and is used extensively as a dietary supplement. Previous studies have shown that the probiotic effects of polysaccharides are closely associated with their molecular properties. The intestinal microbiota has been demonstrated to degrade HA; however, the regulatory effects of different molecular weights (MW) of HA on gut microbiota and metabolites are unknown. In the present study, we performed *in vitro* fermentation of human-derived feces for three MWs of HA (HA1, 32.3 kDa; HA2, 411 kDa; and HA3, 1510 kDa) to investigate the differences in the fermentation properties of HA with different MWs. We found that gut microbiota can utilize all HAs and, consequently, produce large amounts of short-chain fatty acids (SCFAs). In addition, we showed that all three HA MWs promoted the growth of *Bacteroides*, *Parabacteroides*, and *Faecalibacterium,* with HA1 being more effective at promoting the growth of *Bacteroides*. HAs have various regulatory effects on the structure and metabolites of the gut microbiota. Spearman’s correlation analysis revealed that alterations in gut microbiota and their metabolites were significantly correlated with changes in metabolic markers. For instance, HA1 enriched α-eleostearic acid and DL-3-aminoisobutyric acid by regulating the abundance of *Bacteroides*, and HA3 enriched Thymidin by regulating *Faecalibacterium*. Collectively, the fermentation properties of HA vary across MW, and our results provide insights into the potential association between the MW of HA and its fermentation characteristics by the gut microbiota. These findings provide insights into the influence of the gut microbiota and HAs on the health of the host.

## 1. Introduction

Hyaluronan (HA), an endogenous linear glycosaminoglycan, consists of repeating disaccharide units composed of N-acetyl-glucosamine and β-glucuronic acid [1]. HA is a high-molecular-weight (HMW) polymer found in synovial fluid and the extracellular matrix of several tissues [2]. Despite its simple chemical composition, HA plays important roles in tissue remodeling, inflammation, and cancer formation and has been extensively applied in medicine, food, and healthcare [3]. A growing body of evidence has shown that the biological activity of HA is intricately linked to its molecular weight (MW) [4]. HMW-HA (MW ≥ 100 kDa) has good viscoelasticity, moisturizing properties, and promotes wound healing [5]. In contrast, low-molecular-weight HA (LMW-HA) (1–100 kDa) is more readily absorbed by the host than HMW-HA and plays a significant role in the formation of HA cross-linking products and chronic wound healing [6]. In addition, HA oligosaccharides play a crucial role in promoting fibroblast proliferation, inflammatory mediator expression, and tumor growth inhibition [7]. Recent studies have shown that HA has the potential to modulate intestinal barrier function. Multiple MWs of HA have been shown to alleviate dextran sodium sulfate (DSS)-induced colitis [8,9].

Orally administered HA can be degraded into oligosaccharides by the gut microbiota [10] and the genus-level relative abundance of *Bifidobacterium*, *Faecalibacterium*, *Dialister*, and *Bacteroides* can be modulated by HA fermentation [11]. The effect of HA as a popular dietary supplement on the composition of the gut microbiota, as well as degradation and metabolism of HA by the gut microbiota, has become an important topic. The gut microbiota is a highly complex microbial community and it can affect host health, including obesity, metabolic syndrome, and cardiovascular disease, which are associated with alterations in the gut microbiota. Oral administration of HA can improve resistance to pathogenic bacterial infections and inhibit DSS-induced intestinal inflammation in mice by regulating the structure and composition of the gut microbiota, promoting the abundance of *Akkermansia muciniphila*, and increasing short-chain fatty acid (SCFA) levels [12]. These results suggest that oral administration of HA may directly interact with the gut microbiota, increasing the abundance of beneficial bacteria such as *Bifidobacterium* and *Akkermansia muciniphila*, promoting the production of SCFAs, maintaining intestinal homeostasis, and indicating that HA has the potential to be developed as a novel prebiotic. However, the interactions between the gut microbiota and different MWs of HA are still poorly understood, severely hindering the development of HA. Polysaccharides’ biological activity is strongly related to their MW; for example, LMW blackberry polysaccharides are more easily degraded and used by the gut microbiota [13]. Therefore, it is important to determine the fermentation characteristics of HA with different MWs during intestinal digestion.

In this study, we investigated the fermentability characteristics and prebiotic functions of HA with different MWs in six healthy gut microbiota using *in vitro* fermentation. To examine HA degradation, we assessed HA consumption and the amounts of SCFAs. In addition, we applied untargeted metabolomics in combination with 16S rRNA gene high-throughput sequencing to investigate the impact of the three MWs of HA on the modulation of the gut microbiota during metabolism. In conclusion, we investigated whether there are differences in the effect of different MWs of HA on the structure and metabolites of the gut microbiota by *in vitro* fermentation. This information is useful for understanding the relationship between MW and function of polysaccharides and provides guidance for potential prebiotic applications of HA with different molecular weights.

## 2. Materials and Methods

### 2.1. Materials

Hyaluronan (HA1, 32.3 kDa; HA2, 411 kDa; and HA3, 1510 kDa) was generously provided by Topscience Biotech Co., Ltd. (Shandong, China). Standard SCFA reagents were sourced from the China National Medicines Corporation Ltd. (Beijing, China). Ultrapure water was used in all the experiments. The organic reagents were purchased from Thermo Fisher Scientific (Waltham, MA, USA).

### 2.2. Sample Preparation for Stool

Fresh fecal samples were collected from six individuals (three from women and three from men, ages: 22–30 years, BMI: 18.5–24), who had not been prescribed antibiotics or probiotics for at least 3 months and without digestive disease. The study protocol was approved by the Medical Ethics Committee of the Affiliated Hospital of Jiangnan University (approval number: JNU20220901IRB07). All participants provided informed consent before participation. The information on volunteers were shown in Table 1.

Immediately after collection, each fresh fecal sample was homogenized at a ratio of 1:7 (*m*/*v*) in 100 mM sterile phosphate-buffered saline (PBS; 0.1% cysteine, pH 7.4). Sieves (0.4 mm) were used to filter the combinations, and the resulting suspension was transferred to an anaerobic incubator (Whitley DG250 Anaerobic Workstation, Bradford, West Yorkshire, UK) for fermentation.

### 2.3. In Vitro Fermentation

The fermentation process simulated the fermentation of HA in the colon [14]. A commonly used gut microbiota medium was used as the *in vitro* fermentation medium. However, instead of glucose, 5 g/L HA was used as the carbon source [15]. The medium was autoclaved and placed in an anaerobic incubator to maintain an anaerobic state. The 10 mL fermentation culture medium was inoculated with 2 mL stool suspension, and the mixture was then anaerobically maintained at 37 °C. Parallel tests were performed in triplicate. The fermentation broth was collected at 0, 24, and 48 h for subsequent assays.

### 2.4. HA Degradation Analysis

Supernatant of the fermentation broth after being centrifuged at 4 °C (12,000× *g* for 10 min) was used to analysis the degradation of HA. The HA concentration was measured using the carbazole-sulfuric acid method, with glucuronic acid serving as the reference standard [16]. Parallel tests were performed in triplicate. The results are expressed as the average quantity of HA remaining during fermentation versus the control at 0 h.

### 2.5. SCFAs

The concentration of SCFAs was determined using gas chromatography-mass spectrometry (GC-MS) [17]. After centrifugation at 4 °C (12,000× *g* for 10 min), 500 μL of sample supernatant was acidified by adding 20 μL of 10% H_2_SO_4_. Anhydrous ether (1 mL) was added and the mixture was centrifuged at 4 °C (18,000× *g* for 15 min). The resultant supernatant was combined with anhydrous sodium sulfate (0.25 g), left to stand for 30 min, and centrifuged at 4 °C (18,000× *g* for 15 min). Finally, the upper layer was transferred to a gas-phase vial for GC-MS analysis (QP2010 Ultra, SHIMADZU, Kyoto, Japan). Extracts were measured using a Rtx-Wax capillary column with 0.89 mL/min of helium as the carrier gas. The initial oven temperature was 100°C, while the input temperature was 240 °C. Following injection, the oven temperature climbed to 140°C at a rate of 7.50 °C/min, then to 200 °C at a rate of 60 °C/min for three minutes. The following parameters were programmed into the mass spectrometer: temperature of the ion source: 220 °C; temperature of the interface: 250 °C; solvent delay: 2.5 min; *m*/*z* scan range: 2 to 100 [18].

### 2.6. Gut Microbiota Analysis

The fecal samples were centrifuged at 4 °C (12,000× *g* for 10 min) to remove the supernatant. The FastDNA SPIN kit for feces was used to extract bacterial DNA from the sediments. The V3-V4 gene section of 16s rRNA was amplified using the universal primers 341F and 806R. The quality of the extracted DNA was assessed by agarose gel electrophoresis. Following gel detection using the kit (Biomiga, San Diego, CA, USA), the PCR products were recovered. Genomic DNA was accurately quantified using a Nano Photometer N60 Touch [19]. The Illumina MiSeq platform was used for the paired-end sequencing of purified and merged amplicon libraries.

### 2.7. Untargeted Metabolomics Analysis

Culture media were centrifuged at 4 °C (10,000× *g* for 5 min) and 0.1 mL of the resulting supernatants was added to 0.4 mL of organic liquid chilled at −20 °C (methanol: acetonitrile = 1:1). To precipitate the proteins, the mixture was vortexed vigorously, sonicated in an ice bath for 10 min, and then stored at −20 °C for 1 h. After the proteins were precipitated, the samples were centrifuged at 4 °C (15,000× *g* for 15 min). The supernatant was then transferred to a new 1.5 mL centrifuge tube and dried using a rotary evaporator. The dried samples were re-solubilized in 200 μL of solvent (water: acetonitrile = 1:1) and vortexed. After another centrifugation under the same conditions, the supernatant was filtered through a 0.22-μm filter membrane into an injection bottle. To generate quality control (QC) samples, identical volumes of samples were transferred to fresh injection vials and thoroughly blended. An Atlantis Hilic Silica (3.0 μm, 100 × 2.1 mm) was used as sapartation column. The MS was operated in the ESI ionization mode and scanned from 70 to 1050 with a resolution of 70,000. The mobile phases were as follows: Positive mode, consisting of 10 mM ammonium acetate (0.1% formic acid) and ACN:H_2_O at a ratio of 95:5 for phase A and 50:50 for phase B; Negative mode,10 mM ammonium acetate and ACN:H_2_O at a ratio of 95:5 (pH 9.0 with ammonia) for phase A and 50:50 (pH 9.0 with ammonia) for phase B. Compound Discovery 3.1 (Thermo Fisher Scientific, Waltham, MA, USA) was used to analyze raw data files.

### 2.8. Statistical Analysis

All tests were performed in triplicate and data are expressed as mean ± standard deviation (SD). Statistical significance was set at *p* < 0.05 was determined with analysis of variance (ANOVA) tests followed by Tukey’s multiple cormparisons test. Data were analyzed using GraphPad Prism software (version 9.0).

## 3. Results

### 3.1. Degradation of HA

**Figure 1:**
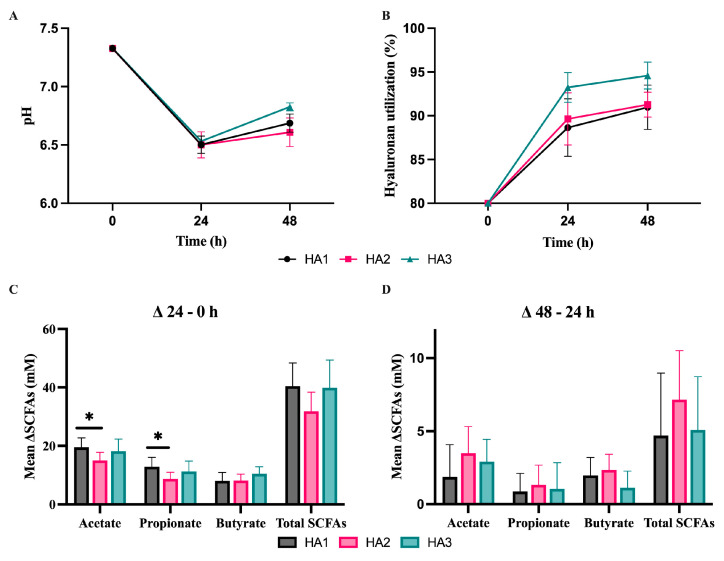
Human feces microbiota fermentation and degradation of HAs *in vitro*. (**A**) pH fluctuations in fermentation medium. (**B**) HAs utilization during fermentation. (**C**) Changes in the SCFAs concentration by HAs fermentation 24 h compared to that at 0 h. (**D**) Changes in the SCFAs concentration by HAs fermentation 48 h compared to that at 24 h. Significant differences were marked based on the *p*-value (* *p* < 0.05).

HA is a new food ingredient that is widely used in the health food industry [20]. However, the poor absorption of HA by oral administration means that its bioavailability is low [21]. HA has previously been shown to not be degraded by gastrointestinal enzymes but can be degraded by the gut microbiota [10]. In the present study, we measured the pH of the fermentation supernatant and determined the HA utilization curve for each sample using the carbazole-sulfuric acid method. There were no significant differences in pH or HA utilization among the fermentation samples of the three sugars. After 24 h of fermentation, the pH values of all samples were at their lowest (Figure 1A) and gradually increased over time. Figure 1B shows the changes in the total HA concentration in the culture media during *in vitro* fecal fermentation. Relative HA content was determined after 24 and 48 h of fermentation. After 24 h, the HA utilization of all three sugars exceeded 85%, and the utilization of HA3 exceeded 90%. After 48 h, HA utilization by the three sugars reached >90%. The six stool samples showed strong HA degradation at all three MWs, as most of the HA was utilized during the first 24 h. This is consistent with the trend of pH change during fermentation. In addition, the amount of HA utilization increased as the MW increased, and we hypothesized that the gut microbiota in the collected stool samples preferred to utilize HMW-HA.

### 3.2. SCFAs Production during Fermentation

SCFAs are fatty acids with fewer than six carbons in their aliphatic tails, and the three most abundant carbons in the intestine are acetate (C2), propionate (C3), and butyrate (C4) [22]. SCFAs are produced by the anaerobic intestinal microbiota by saccharolytic fermentation of complex carbohydrates (for example, dietary fiber, resistant starch, and inulin) that cannot be digested and absorbed by the small intestine [23]. Figure 1C,D show the SCFA variables every 24 h during the fermentation process. The total SCFAs concentration increased substantially after 24 h of fermentation, with an increase of 30–40 mM. By 48 h of fermentation, only HA2 produced a significant amount of SCFAs, which was much less than the 24 h yield. From 24 to 48 h, the production of SCFAs decreased, as most of the HA was utilized in the first 24 h. This is consistent with the trend of HA utilization during fermentation. The main components of SCFAs produced by the anaerobic fermentation of HA *in vitro* include acetate, propionate, and butyrate. During fermentation for 24 h, HA1 produced significantly (*p* < 0.05) higher levels of acetate and propionate than HA2, whereas there was no significant difference in the level of butyrate among the three HA MWs. Studies have shown that SCFAs can maintain intestinal homeostasis, prevent obesity, and ameliorate colitis and diabetes [24,25,26]. Oral acetate improves glucose tolerance [27], whereas butyrate and propionate could prevent diet-induced obesity and regulate intestinal hormones [28]. In summary, all three MWs of HA promoted the production of SCFAs, and the effect of MW was mainly reflected in the levels of acetate and propionate. Small-molecular-weight HA produced comparable or slightly more SCFAs than larger molecules, which is consistent with previously reported results [29].

### 3.3. Changes in Microbial by Fermentation of HA

**Figure 2:**
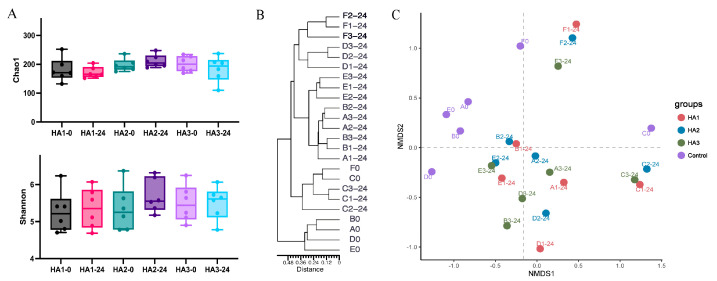
Analysis of microbial diversity in samples following HAs fermentation (**A**) Alpha-diversity analysis, (Chao1 and Shannon indexes). (**B**) Clustering analysis of gut microbiota. (**C**) NMDS.

Human microbiota comprises trillions of microorganisms, with bacteria being the most abundant [30]. The gut microbiota is significantly associated with numerous physiological processes in the host, such as intestinal epithelium and immune system homeostasis [31,32]. It affects the metabolic system and may influence the liver, inflammation, and the cardiovascular system [33]. The consumption of specific dietary components, such as polysaccharides, can serve as a means to modulate the microbial community’s structure and metabolic activity, thereby maintaining intestinal homeostasis [34]. High-throughput 16S rRNA gene sequencing technology was employed to analyze the microbiota structure of fecal samples both pre- and post-fermentation to investigate the interaction between different MWs of HA and the gut microbiota. As most of the HA was degraded at 24 h, the microbiome profiles at 24 h of fermentation were assessed. The Chao1 and Shannon indices were used to estimate community diversity. After 24 h of fermentation, the alpha diversities of HA1, HA2, and HA3 were not significantly different from that of the control group (Figure 2A). Cluster analysis based on evolutionary trees and non-metric multidimensional scaling (NMDS) were used to analyze global structural alterations in the intestinal microbiota before and post-24 h of HA fermentation (Figure 2B,C). Figure 2C shows an obvious taxonomic difference in the microbiota between HAs and control groups at the genus level. In each individual, a distinct separation was observed between HA1, HA2, and HA3. However, due to inter-individual variation in the quantity and diversity of the gut microbiota, no significant differences were observed between HA1, HA2, and HA3. In addition, the distance between the control and HA1 groups was greater, indicating that HA1 significantly influenced the intestinal microbiota.

**Figure 3:**
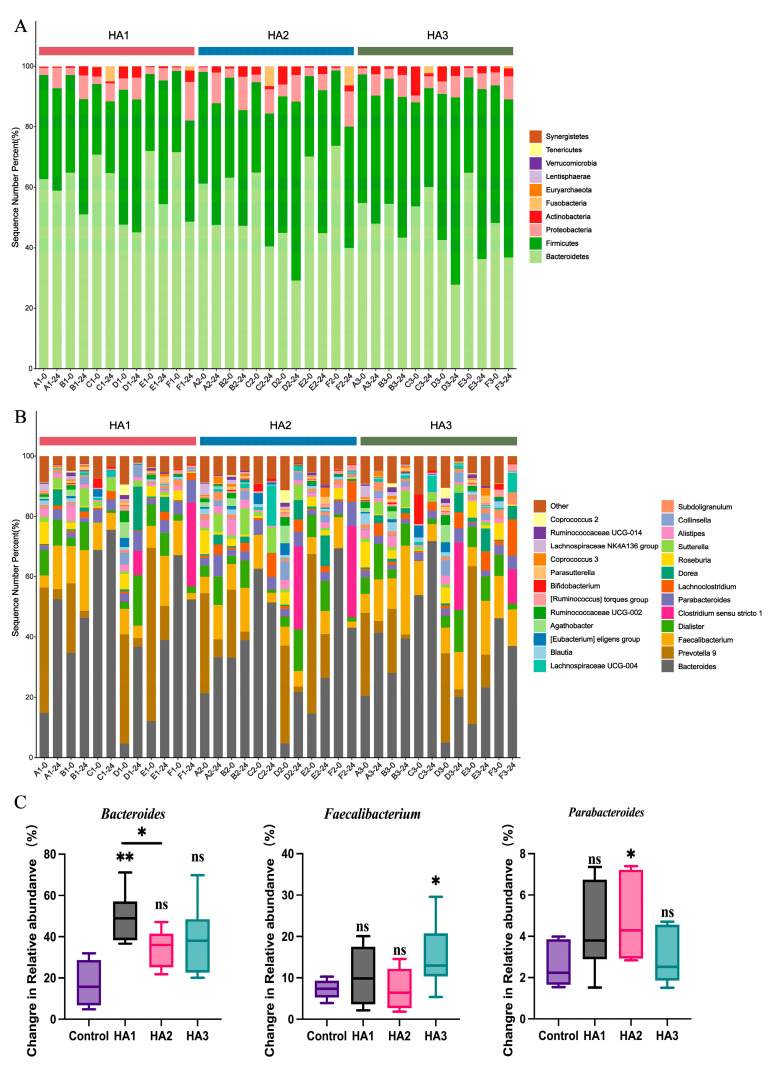
Reaction of the gut microbiota following HA fermentation. Bacterial taxonomic profiling of gut bacteria at the phylum (**A**) and genus (**B**). Change in relative abundance of (**C**) *Bacteroidetes*, *Faecalibacterium*, and *Parabacteroides* by HA fermentation after 24 h compared to after 0 h. Significant differences were marked based on the *p*-value (* *p* < 0.05, ** *p* < 0.01). “ns” means “no significant differences”.

Alterations in the gut microbiota were examined at the genus level following HA fermentation (Figure 2C). At the genus level, the fermentation of HA of all MWs led to an increase in the abundance of *Bacteroidetes*, *Faecalibacterium* and *Parabacteroides*. Previous studies have shown a consistent conclusion; Pan et al. [11] found that fermentation with 1.3 MDa HA increased the frequency of *Bacteroides* and *Faecalibacterium*. These discrepancies may be due to variations in the composition of the medium and gut microbiota used. In addition, it can be concluded from Figure 3C that HA1, HA2, and HA3 are more prominent in promoting the growth of *Bacteroides*, *Parabacteroides*, and *Faecalibacterium* respectively after 24 h fermentation. It has been reported that *Bacteroides* is known to produce acetate and propionate, and *Faecalibacterium* is butyrate producing bacteria [35,36,37,38]. This could explain the differences in the SCFA levels between HA1, HA2, and HA3 after 24 h of fermentation. In addition, structural differences in the gut microbiota of HA1, HA2, and HA3 at the genus level are shown in Figure S1, and 15 differential genera were identified. These findings indicate that the intestinal microbiota is capable of fermenting HA with different MWs. Different MW of HA have various regulatory effects on the structure of the microbiota.

### 3.4. Changes in the Fecal Metabolism

**Figure 4:**
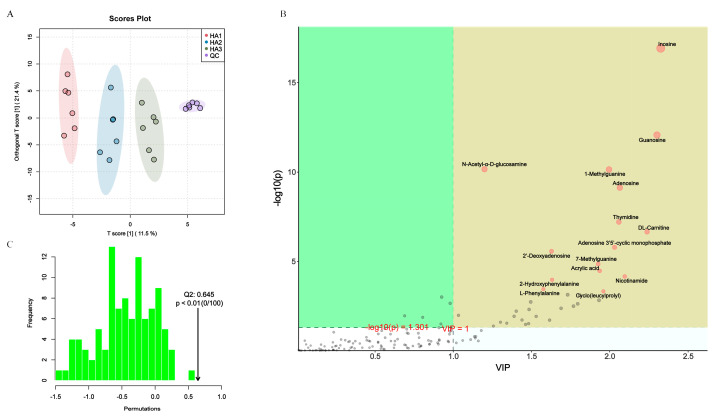
Alterations in metabolism caused by HAs fermentation. (**A**) PCA. (**B**) OPLS-DA. (**C**) OPLS-DA score plot.

Metabolomics is the analysis of small molecular intermediates and end products in biological samples that provide a valuable characterization of the biological profile and reflect the metabolism of the colony [39]. In this study, untargeted metabolomics of the fermentation broth supernatant was used to investigate the modulatory effects of the three MWs of HA on the metabolite profiles of the intestinal microbiota. The metabolite profiles were evaluated after 24 h of fermentation, and 146 active substance peaks were detected. In addition, the similarity of the principal components and overall metabolic differences between the sets of samples were examined using principal component analysis (PCA; Figure 4A) and orthogonal partial least squares discriminant analysis (OPLS-DA; Figure 4C). The PCA score plot showed that the QC group clustered together, signifying consistent instrument performance and minimal reliable data errors, whereas the HA1, HA2, and HA3 groups showed significant differences. This result further demonstrates that different MWs of HA have various modulatory effects on microbiota composition as well as the profiles of microbiota metabolites. The accuracy of the prediction was 0.645 (Q2), which satisfied the requirements for a precise prediction. (Figure 4B). Similar studies demonstrated that different MWs of guar gum have different abilities to regulate bile acid levels in the cecum [40].

**Figure 5:**
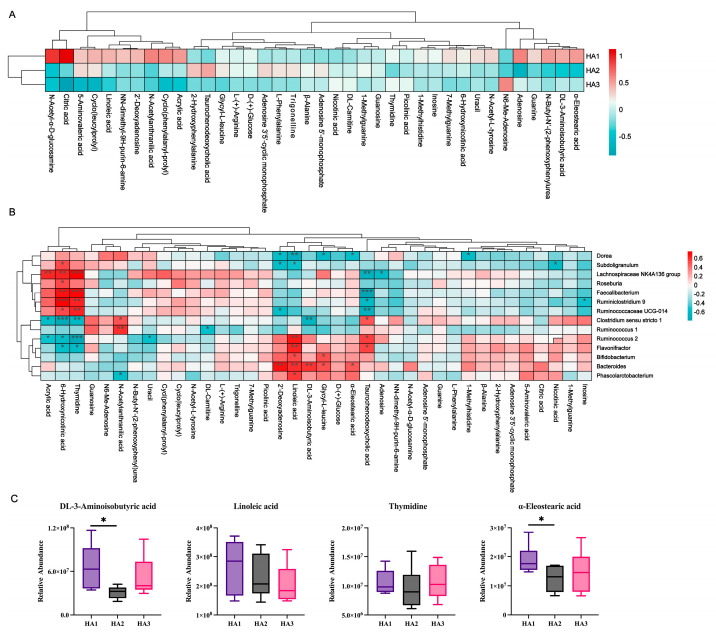
(**A**) Heat map of differential metabolite clustering among the groups after HAs fermentation. (**B**) Correlation analysis between the altered gut microbiota and metabolites. (**C**) Relative abundance of DL-3-aminoisobutyric acid, linoleic acid, thymidine, and α-eleostearic acid after 24 h of HA fermentation. Significant differences were marked based on the *p*-value (* *p* < 0.05, ** *p* < 0.01, *** *p* < 0.001.).

Molecular properties of polysaccharides affect microbiota metabolism. Thus, there is the potential for different MWs of HA to target the modulation of the intestinal microbiota metabolome. By further screening the matches based on the VIP values obtained from OPLS-DA (red dots indicate VIP > 1), the metabolites with VIP > 1 were selected as potential biomarkers (Figure 4B). Overall, 38 variables were identified as biomarkers (Table S1), which serve as characteristic biomarkers with high intragroup confidence. Metabolomic analysis, as shown in Figure 5A, revealed that HA1 increased the content of N-acetyl-α-D-glucosamine, citric acid, 5-aminovaleric acid, Cyclo(leucylprolyl), linoleic acid, N,N-dimethyl-9H-purin-6-amine, 2’-deoxyadenosine, N-acetylanthranilic acid, cyclo(phenylalanyl-prolyl), acrylic acid, adenosine, guanine, N-Butyl-N’-(2-phenoxyphenyl)urea, DL-3-aminoisobutyric acid, and α-eleostearic acid. HA2 increased the content of 5-aminovaleric acid, cyclo(leucylprolyl), 2-hydroxyphenylalanine, taurochenodeoxycholic acid, and adenosine 3′5′-cyclic monophosphate. HA3 increased the content of N6-Me-Adenosine.

### 3.5. Association of Gut Microbiota and Metabolites

The influence of the gut microbiota on the host is intricately linked to the microbial metabolic axis. Previous studies have shown that *Poria cocos* oligosaccharides ameliorate the development of glucolipid metabolism disorders in HFD-fed mice, and correlation analyses have demonstrated that changes in the gut microbiota and metabolites induced by *Poria cocos* oligosaccharides are significantly associated with changes in metabolic markers [41]. To elucidate the link between microbes and metabolites, Spearman’s correlation analysis was performed on the significantly altered microbiota taxa and metabolites after the fermentation of the three HAs. The correlation analysis of the 15 gut bacteria that differed in the three HAs with 38 altered metabolites is shown in Figure 5B. The α-eleostearic acid, DL-3-aminoisobutyric acid, and linoleic acid levels were strongly positively correlated with the abundance of *Bacteroides*, and Thymidine levels were strongly positively correlated with the abundance of *Faecalibacterium*. The levels of α-eleostearic acid and DL-3-aminoisobutyric acid were significantly higher in HA1 than in HA2, providing further evidence that the levels of these metabolites corresponded to the abundance of *Bacteroides* in the samples (Figure 5C). The present study suggests that HA of different MWs can further influence bacterial community metabolites by affecting the structure of intestinal colonies. Meanwhile, DL-3-aminoisobutyric acid can reduce liver inflammation in obese mice and hepatocyte apoptosis in diabetic mice [42,43], and can function as a protective metabolite in the intestine to alleviate Clostridium difficile infection in mice [44]. α-eleostearic acid reduces the severity of mice with inflammatory bowel disease by stimulating the peroxisome proliferator-activated receptor-γ [45]. Chrysanthemum polysaccharides and luteolin ameliorate colitis in rats by modulating linoleic acid and purine metabolism [46,47].

A recent study showed that HA (34 KDa) induces alterations in the intestinal microbiome and protects mice from *Citrobacter rodentium* infection and intestinal inflammation [12]. We speculate this MW of HA attenuates intestinal inflammation in pathogenic and chemically induced intestinal mucosal injury by modulating the levels of DL-3-aminoisobutyric acid as well as α-eleostearic acid in intestinal colony metabolites. Studies have also reported that the therapeutic effects of chitosans on ulcerative colitis vary by MW [48], as they exhibit variations in antioxidant, anti-inflammatory, immunoglobulin modulation, and intestinal microbiota regulation. In conclusion, HA of different MWs can target the regulation of intestinal microbiota and metabolites, and differences in MW may cause different therapeutic effects in diseases such as colitis. These findings shed light on the effects of MW on the prebiotic potential of HA *in vitro* and contribute to its future use as a functional food. The conclusions of this study are based on an *in vitro* fermentation system, which is farthest from physiological conditions and needs to be further validated by clinical trials.

## 4. Conclusions

In conclusion, an *in vitro* fermentation procedure was used to investigate the interaction between HA of different MWs and the intestinal microbiota. These findings suggest that, under anaerobic conditions in the colon, HMW-HA is more readily degraded by the gut microbiota, whereas LMW-HA produces a higher amount of SCFA. Different MWs of HA were fermented by the microbiota in human feces and differed in their effects on the microbiota, metabolites, and metabolic pathways. HA1 can increase the abundance of α-eleostearic acid and DL-3-aminoisobutyric acid promoting the growth of *Bacteroides*, and HA3 can increase the level of Thymidin by promoting the growth of *Faecalibacterium*. Differences in fermentation properties due to MW confer more possibilities for the development of HA products. Our research provides the scientific basis for the targeted regulation of gut microbiota by oral HA. In addition, oral HA1 as well as HA3 may have the potential to attenuates intestinal inflammation, which would facilitate the development of new therapies for many critical diseases with broad and important public health implications.

## Figures and Tables

**Table 1:** Information on volunteers.

Volunteers	Gender	Age	BMI
A	Female	24	21.5
B	Female	26	22.7
C	Female	25	20.2
D	Male	24	21.8
E	Male	30	21.8
F	Male	22	23.2

## Supplementary Materials

The following supporting information can be downloaded at: https://www.mdpi.com/article/10.3390/polym15092103/s1, Figure S1: Using the random forest method, the highest contributing features of gut microbiota were selected; Table S1: Totally 38 metabolites were identified as biomarkers with large between-group differences, which represent characteristic biomarkers with high intra-group confidence.
