# Supplementary material for: In Vitro Fermentation of Hyaluronan with Different Molecular Weights by Human Gut Microbiota: Differential Effects on Gut Microbiota Structure and Metabolic Function
Source: Polymers (Basel). 2023 Apr 28;15(9):2103. doi: 10.3390/polym15092103 (PMC10180753; doi:10.3390/polym15092103)
Supplement: Supplementary file 1 [file polymers-15-02103-s001.zip › Table S1.pdf]

Table S1. Totally 38 metabolites were identified as biomarkers with large between-group differences, which represent characteristic biomarkers with high intra-group confidence.

No.	Compound name	Chemical Formula	VIP score
1	Inosine	C10 H12 N4 O5	2.33
2	Guanosine	C10 H13 N5 O5	2.30
3	DL-Carnitine	C7 H15 N O3	2.24
4	Nicotinamide	C6 H6 N2 O	2.10
5	Adenosine	C10 H13 N5 O4	2.07
6	Thymidine	C10 H14 N2 O5	2.06
7	Adenosine 3′5′-cyclic monophosphate	C10 H12 N5 O6 P	2.03
8	1-Methylguanine	C6 H7 N5 O	2.00
9	Cyclo(leucylprolyl)	C11 H18 N2 O2	1.96
10	Acrylic acid	C3 H4 O2	1.94
11	N6-Me-Adenosine	C11 H15 N5 O4	1.93
12	7-Methylguanine	C6 H7 N5 O	1.93
13	D-(+)-Glucose	C6 H12 O6	1.81
14	Cyclo(phenylalanyl-prolyl)	C14 H16 N2 O2	1.80
15	Adenosine 5'-monophosphate	C10 H14 N5 O7 P	1.77
16	5-Aminovaleric acid	C5 H11 N O2	1.73
17	L-(+)-Arginine	C6 H14 N4 O2	1.69
18	2-Hydroxyphenylalanine	C9 H11 N O3	1.63
19	2′-Deoxyadenosine	C10 H13 N5 O3	1.63
20	Citric acid	C6 H8 O7	1.62
21	L-Phenylalanine	C9 H11 N O2	1.58
22	NN-dimethyl-9H-purin-6-amine	C7 H9 N5	1.53
23	Glycyl-L-leucine	C8 H16 N2 O3	1.50
24	β-Alanine	C3 H7 N O2	1.48
25	DL-3-Aminoisobutyric acid	C4 H9 N O2	1.48
26	N-Acetylanthranilic acid	C9 H9 N O3	1.46
27	Picolinic acid	C6 H5 N O2	1.40
28	Trigonelline	C7 H7 N O2	1.39
29	N-Acetyl-L-tyrosine	C11 H13 N O4	1.34
30	Uracil	C4 H4 N2 O2	1.26
31	N-Acetyl-α-D-glucosamine	C8 H15 N O6	1.20
32	1-Methylhistidine	C7 H11 N3 O2	1.18
33	N-Butyl-N′-(2-phenoxyphenyl)urea	C17 H20 N2 O2	1.16
34	6-Hydroxynicotinic acid	C6 H5 N O3	1.14
35	Linoleic acid	C18 H32 O2	1.14
36	Guanine	C5 H5 N5 O	1.11
37	Taurochenodeoxycholic acid	C26 H45 N O6 S	1.03
38	α-Eleostearic acid	C18 H30 O2	1.02
