# Supplementary figures and images for: In Vitro Fermentation of Hyaluronan with Different Molecular Weights by Human Gut Microbiota: Differential Effects on Gut Microbiota Structure and Metabolic Function
Source: Polymers (Basel). 2023 Apr 28;15(9):2103. doi: 10.3390/polym15092103 (PMC10180753; doi:10.3390/polym15092103)

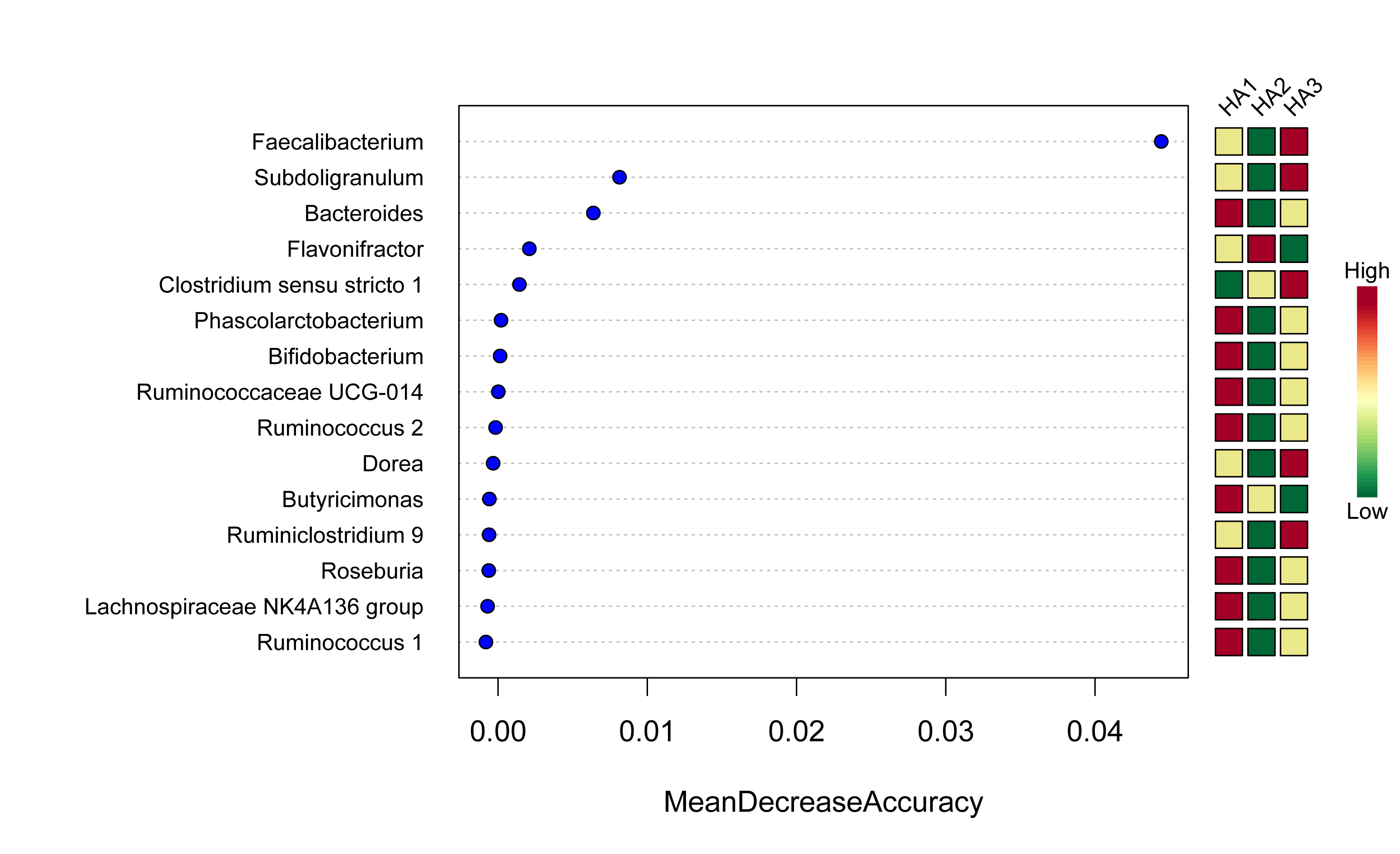

Supplement: Supplementary file 1 [file polymers-15-02103-s001.zip › Figure S1.tif]
